# Tracking Inhibitory Control in Youth With ADHD: A Multi-Modal Neuroimaging Approach

**DOI:** 10.3389/fpsyt.2020.00831

**Published:** 2020-11-19

**Authors:** Lescia K. Tremblay, Christopher Hammill, Stephanie H. Ameis, Mehereen Bhaijiwala, Donald J. Mabbott, Evdokia Anagnostou, Jason P. Lerch, Russell J. Schachar

**Affiliations:** ^1^ Department of Neurosciences and Mental Health, Hospital for Sick Children, Toronto, ON, Canada; ^2^ The Margaret and Wallace McCain Centre for Child, Youth & Family Mental Health, Campbell Family Mental Health Research Institute (CAMH), Toronto, ON, Canada; ^3^ Department of Paediatrics, Holland Bloorview Research Institute, Toronto, ON, Canada

**Keywords:** attention deficit hyperactivity disorder (ADHD), diffusion tensor imaging (DTI) tractography, inhibitory control, youth, stop signal task

## Abstract

**Background:**

A decreased ability to inhibit a speeded motor response is a well-studied deficit in Attention Deficit Hyperactivity Disorder (ADHD), and has been proposed as an endophenotype. Inhibitory control has been assessed reliably with the Stop Signal Task (SST) and is associated with prior documented differences in regional brain function using f-MRI. Here, we advance on these findings by examining their structural connectivity and white matter integrity with the goal of identifying a network underlying a core cognitive deficit in ADHD.

**Methods:**

Healthy controls (N=16) and youth diagnosed with ADHD (N=60) were recruited through the Province of Ontario Neurodevelopmental Disorders Network (POND) and the Hospital for Sick Children. An f-MRI activation difference map was co-registered with each participant’s white matter imaging data, representing the specific network nodes where ADHD youth diverged significantly from controls while performing the SST. Probabilistic tractography was applied from these nodes, and white matter integrity indices such as fractional anisotropy (FA) within the tracts of interest were contrasted between the groups and correlated with SST output measures, including the measure of inhibitory control, the stop signal reaction time (SSRT).

**Results:**

The tracts that connected the network nodes belonged primarily to the inferior fronto-occipital fasciculus (IFOF) and cingulum. ADHD subjects showed trend differences in FA compared to controls between right inferior frontal gyrus (IFG) and right superior temporal gyrus (P= 0.09), right IFG and right posterior cingulate (P= 0.01), right anterior cingulate to posterior cingulate (p= 0.08), and between left middle temporal gyrus (BA 39) and left posterior cingulate (P=0.02). A trend correlation was found between radial diffusivity within IFG to STG white matter (IFOF) and SSRT.

**Conclusions:**

We identified potential white matter tracts related to deficient inhibitory control, elucidating the brain mechanisms of an important cognitive deficit in ADHD. These findings could be integrated into future endophenotypic biomarker studies, incorporating altogether brain structure, function, and behavior for future studies of ADHD and other psychiatric conditions that exhibit this deficit.

## Introduction

Attention Deficit Hyperactivity Disorder (ADHD) is a childhood onset neurodevelopmental disorder with a prevalence estimate of 5% (1), characterized clinically by elevated activity levels, inattentiveness, and high impulsivity. Children with ADHD often exhibit difficulties with peer relationships, poor functioning at school, and increased psychiatric comorbidity (2). Etiological studies implicate polygenic and environmental mechanisms that impact neurodevelopment, and that have recently been shown to overlap with other associated traits, such as decreased educational attainment, tobacco dependence, depression, and sleep disturbance (3). Structural and functional imaging studies of ADHD have revealed differences spanning grey and white matter of all cortical lobes and many subcortical structures (4, 5) suggestive of a distributed brain network disturbance, rather than a disorder with discrete regional abnormalities (6, 7). Diffusion Tensor Imaging (DTI) of white matter connectivity in ADHD has also shown broad white matter changes within nearly all major tracts (8, 9).

With this extensive background of neuroimaging findings, it would be advantageous to link these with key behavioral processes, such as executive functioning in ADHD (10, 11). These could potentially map more reliably to brain structure and function compared to clinical symptoms listed within the DSM-5 (12). Shifting away from symptom criteria is in line with the Research Domain Criteria-RDoc method, yielding potential insights into specific brain network changes related to important neurocognitive constructs in ADHD neurobiology (13, 14).

Here, we take advantage of a well replicated cognitive deficit in ADHD, poor inhibitory control, to specifically probe underlying white matter connectivity changes. Inhibitory control is an executive function that encompasses different cognitive components including prospective withholding, which is preparing to withhold a response such as a button press versus appropriately pressing down, and reactive inhibition, which is stopping a response quickly, and error detection (15–17). Contrary to symptoms that are often variable throughout development and psychosocial context (e.g. classroom versus home environments), and that yield a diagnosis that is heterogeneous in severity and symptom clusters (e.g. hyperactive vs. inattentive types), poor inhibitory control is stable over time, is shown to persist in remitted and non-remitted ADHD, and thus provides us with a more state-independent behavioral marker (18).

Inhibitory control has often been measured with the stop signal task (SST) (19, 20), and has been applied successfully with functional neuroimaging previously (16, 21, 22). The SST provides a laboratory analogue of real life situations wherein one must respond as quickly and as accurately as possible to trials involving a simple choice response while remaining vigilant for intermittent signals that indicate that one’s response has to be withheld. The SST provides us with a measure of latency of stopping, termed the stop signal reaction time (SSRT). A longer (i.e. slower) SSRT in ADHD subjects is a well-replicated finding (20), with a medium effect size of 0.63 (23), and modifiable with ADHD medications including methylphenidate (24, 25). It is also demonstrable in siblings and parents (26, 27), and associated with polymorphisms in dopamine genes (28). Based on this body of evidence, it has been described as a candidate endophenotype (29) and represents therefore an excellent tool to better understand altered brain mechanisms in ADHD.

In a past f-MRI study with the SST, we investigated where ADHD youth differed from healthy controls and found that activation differed mainly within the time where prospective withholding occurred (30). Within that same study, a pilot study was conducted applying Diffusion Tensor Imaging (DTI) with probabilistic tractography to explore connectivity between these identified regions (i.e. network nodes), providing us with white matter tracts of interest underlying the inhibitory control deficit, which included the inferior fronto-occipital fasciculus (IFOF) connecting fronto-temporal nodes. A trend correlation between disturbed white matter integrity and the degree of the inhibitory deficit was also found within this tract (31). In the current study, our multimodal approach, which triangulates brain structure, function, and a cognitive deficit, was expanded to a larger pediatric dataset with available DTI data, from which we applied probabilistic tractography. Probabilistic tractography is a tracking algorithm that applies a Bayesian model to estimate the probability of connectivity between groups of voxels representing regions of interest (i.e. “seeds”) using water diffusion measures, enabling visualization of structural connectivity. From these tracts, DTI-derived metrics of white matter microstructure (32, 33) are calculated, including fractional anisotropy (FA), which is a measure of the total magnitude of water diffusion along the axonal fibers. Changes in FA are associated with an overall disruption in white matter, and often represents the primary outcome measure of white matter integrity in DTI studies. Secondary metrics are radial diffusivity (RD) and axial diffusivity (AD), where increased RD and AD are thought to provide indices of disturbed myelination (34) and axonal structure (35) respectively, however, this remains under continuous study (36). We applied a relatively novel approach for seeding for the tractography, that is, instead of using predefined manually-drawn seeds to extract a pathway of interest, guided by techniques described in recently developed DTI atlases (37, 38), we incorporated functionally-informed seeding (i.e. the F-MRI brain activity difference map) into diffusion imaging to probe potential structural white matter changes related specifically to inhibitory control in ADHD. This brain structure-function multimodal approach helped circumvent the important challenge of having to manually draw in tractography seeds *a priori* within complex structures like the prefrontal cortex, particularly in the developing brain, where one designated region can have varying structural topographical labels (e.g. inferior frontal gyrus/IFG vs. ventro-lateral prefrontal cortex vs. Brodmann Areas 44 to 47). Both anatomical tracer studies and functional connectivity MRI studies reinforce strongly that within higher order structures such as the prefrontal cortex and association cortex overall, two closely adjacent regions are connected to multiple and very distinct distributed networks (39, 40). And, in a meta-analysis of inhibitory control measured by various inhibitory control tasks in healthy individuals, activation within the IFG alone spanned between the middle frontal gyrus, frontal operculum, and anterior insula (41).

Our goal was to further elucidate potential alterations within the network of white matter tracts relevant to the inhibitory control deficit in children with ADHD, which would lead to further characterization of an important potential endophenotype for future studies of ADHD neurobiology. We hypothesized that white matter alterations would occur between those regions underlying inhibitory control performance, and, that white matter integrity would influence the degree of inhibitory deficit measured by the SST within specific tracts including fronto-temporal white matter within the IFOF.

## Materials and Methods

### Participants

A total of 77 participants took part in the study, however poor diffusion data led to the exclusion of a number of subjects, resulting in N=60 ADHD and N=16 controls. Informed consent was obtained from all individual participants. Participants were recruited through the Province of Ontario Neurodevelopmental Network (POND), Hospital for Sick Children site. For the ADHD group, participants received a consensus diagnosis of ADHD following a multi-disciplinary and comprehensive assessment within a specialized ADHD assessment clinic, involving parent and child interviews using the Parent Interview for Child Symptoms (P.I.C.S.) (42), followed by a Psychiatric consultation. Full-scale IQ was measured by a trained psychometrist or psychologist with age-appropriate Weschler or Stanford Binet scales. The inclusion criteria were: 10-18 years of age, either sex, DSM-V diagnosis of ADHD with possible co-morbid Oppositional Defiant Disorder (ODD), Conduct Disorder (CD) and reading disorder only (no other Axis I co-morbidities), at least moderate impairment in two settings (home, school) scoring below 60 on the Global Assessment Scale (GAS) (43), IQ > 80, no serious medical illness or sensory deficit, and no contraindications for an MRI. The severity of ADHD symptoms was measured with the Strengths and Weaknesses of Attention-Deficit/Hyperactivity-symptoms and Normal-behaviors (SWAN) Scale (44). SWAN scores range from -54 to 54, show good test-retest reliability, and converge with clinical diagnoses of ADHD (45). We calculated SWAN Inattentive (inattentive) and SWAN Hyperactive-impulsive (hyperactive) sub-scores for potential correlation calculations with neuroimaging measures. Medication history was documented, including medications prescribed and taken in the last six months. Participants were asked to withhold ADHD medication (psychostimulant, atomoxetine, and/or guanfancine) 24 hours prior to the session, which occurred in all with the exception of four subjects due to parental concerns regarding their child’s functioning for the rest of the day (e.g. for return to school after study session).

Control subjects aged 10-18 were recruited mainly for the diffusion imaging (DTI) component through internal hospital postings or word of mouth. They were assessed in a comparable manner (i.e. including IQ testing and SWAN scale administration) and reported no psychiatric or medical disorders, including no developmental diagnosis, and no first-degree family history of such.

### The Stop Signal Task (SST)

The SST was administered to all ADHD subjects prior to neuroimaging. The SST estimates the speed with which a person voluntarily executes and stops a speeded motor response, described previously (19). Participants respond as quickly as possible to trials of visual stimuli by pressing a game pad button after seeing a letter “X”(with left thumb) or “O” (with right thumb), which represents the GO phase of the task, interspersed randomly with trials containing a stop signal, where an auditory stimulus is heard at various times and following the time the “X” or “O” occurs, requiring one to withhold from pressing the button, representing the STOP phase of the task. The stop signal occurred in 33% of the trials, delayed initially by 250 ms, then adjusted subsequently from there depending on if the subject failed (delay < 50 ms) versus successfully stopped (delay > 50 ms). There were 4 experimental blocks and 1 practice block, with 24 trials per block. The task paused after each block allowing for a short rest. The total administration time was approximately 10 minutes. Further description is documented by Bhaijiwala et al. (46). We calculated the overall reaction time and the latency of stopping, termed the Stop Signal Reaction time (SSRT). Shorter (i.e. faster) SSRT represents superior inhibitory control. The SSRT is estimated by subtracting the mean delay on the Stop signal trials from the mean reaction time on the no-Stop trials (19, 47). Inhibitory control measured by the SSRT requires a balance between proper withholding of responses (i.e. prospective withholding), that occurs throughout the GO and STOP phases of the task, and fast reaction to stop signals (i.e. reactive inhibition).

### Diffusion Tensor Imaging Protocol (DTI)

Brain imaging was acquired with a 3T MRI (Siemens,TimTrio, Malvern, Pa.) system at the Hospital for Sick Children, using a 12-channel head coil. Anatomical scans were acquired using a three-dimensional T1-weighted MPRAGE sequence (field of view=192 x 240 x 256 mm, 1 mm cubic voxels, time to repeat/echo time/TI=2,300 ms/2.96ms/900 ms, fractional anisotropy=9°, GRAPPA=2). Diffusion data were acquired using a two-dimensional diffusion-weighted echoplanar imaging sequence (axial, field of view= 244 x 244 mm, 70 interleaved 2-mm thick slices, 232 mm2 in-plane resolution, time to repeat/echo time=8,800 ms/87 ms, GRAPPA=2, b=1,000 seconds/mm2, 60 directions). FSL software (Oxford, UK): MRIB’s Diffusion Toolbox: http://fsl.fmrib.ox.ac.uk (version 5.0.9) was used to process these data, including removal of non-brain tissue, correction of head movement and eddy currents, rotation of the B-matrix (48), and to obtain key diffusion parameters for each voxel (49) including eigen(λ) -values λ1 (axial diffusivity), λ2, λ3. Based on what we found in our previous pilot study (31) using these different parameters, we identified fractional anisotropy (FA) as our primary outcome measure, and secondary measures were radial diffusivity (RD= (λ2 + λ3)/2) and axial diffusivity (AD).

### Importing f-MRI-Stop Signal Task Brain Maps for Tractography

The f-MRI brain map of choice that was used as seeds for tractography (described later) was a group contrast map from our prior study (30), containing statistically significant brain regions where youth with ADHD diverged from controls while performing the Stop Signal Task. The deviation in brain activation was most pronounced (i.e. as per the number of brain regions) during the prospective withholding phase of the task, with fewer during the reaction inhibition phase. Seeds of both phases are detailed later in the Results section within **Table 2**. Seed regions were registered to MNI space using the ANTs package (http://stnava.github.io/ANTs/). Since these were derived from functional brain imaging, there would not necessarily be direct structural connectivity (e.g. IFG to ACC) within our brain map, therefore we added two waypoints with previously established structural as well as functional connectivity to our original nodes, that is, the posterior cingulate (50, 51) and caudate (52). Although these waypoints do not represent where differential activation occurred between ADHD versus controls, these activated during the SST in both groups (30), and represent important functional network nodes implicated in ADHD pathophysiology (53).

### Probabilistic Tractography

Seeds were submitted to the FSL-FDT probabilistic tractography algorithm [https://fsl.fmrib.ox.ac.uk, version 5.0.9 (54)] for each subject. The individual tractography was performed on the diffusion tensors in MNI space. The tractography algorithm generates connectivity distributions between the seeds, sampling 5000 potential sample tracts every 0.5 mm, spanning 80 degrees in curvature, retaining tracts that pass through at least one of the other seeds. Because of the breadth of connectivity from the f-MRI derived brain maps spanning multiple lobes bilaterally, probabilistic tractography was done by seed pairing (e.g. IFG to STG), separately for the right and left hemisphere, yielding eleven tracts in the left hemisphere, and eight tracts in the right hemisphere. For the required waypoints we used the structure segmentation from the AAL atlas (55) as a waypoint mask and then binarized and dilated these by three voxels to ensure overlap with surrounding white matter. Both waypoints were used to connect seeds of prospective withholding and reactive inhibition phases.

### White Matter Structural Indices and Correlations With Symptoms and Inhibitory Control

Fractional anisotropy (FA), axial diffusivity (AD), and radial diffusivity (RD) were extracted from probabilistic tractography findings using the FSL software output. For tract thresholding (i.e. to minimize false-positive streamlines), we used a validity cutoff of 150 streamlines passing through a voxel. The SPSS^®^ software program was used to perform t-tests contrasting these white matter measures between controls and ADHD, within all the tracts (i.e. 11 tracts in the left hemisphere and 8 in the right hemisphere). The Benjamini-Hochberg False Discovery Rate (FDR) was applied for multiple-comparison corrections.

The tracts where ADHD differentiated from controls represented our tracts of interest for correlation analyses involving the behavioral measures. Correlations were performed between SWAN scale scores, that is, both hyperactivity-impulsivity and inattention scores, with white matter indices (FA, RD, AD) using Pearson’s coefficient. Correlations between the same white matter indices and inhibitory control (SSRT) were also performed for these specific tracts. Although we were only interested in the correlations implicating those tracts where ADHD differentiated from the controls, the Benjamini-Hochberg False Discovery Rate (FDR) multiple-comparison testing was done for the correlations considering all tracts. Finally, SWAN scale hyperactivity-impulsivity and inattention scores were correlated with SSRT.

## Results

See **Table 1** for subject characteristics.

**Table 1:** Subject characteristics.

	**ADHD (N=60)**	**Controls (N=16)**	**Sig.**
**Age**	10.6 (sd=2.6)	10.5 (sd= 4.86)	P=0.941
**Sex (M/F)**	50/10	5/11	P=0.001
**Family History of ADHD (%)**	46%	–	–
**ADHD Type**	72% Combined 21% Inattentive 7% Hyperactive	–	–
**SWAN score**	Inattention score = 5.8 Hyperactivity score = 4.2	–	–
**ADHD Medication History in the last 6 months (yes/no)**	28/32	–	–
**Full Scale IQ**	101	111	P= 0.16

### Probabilistic Tractography Findings

**Figure 1:**
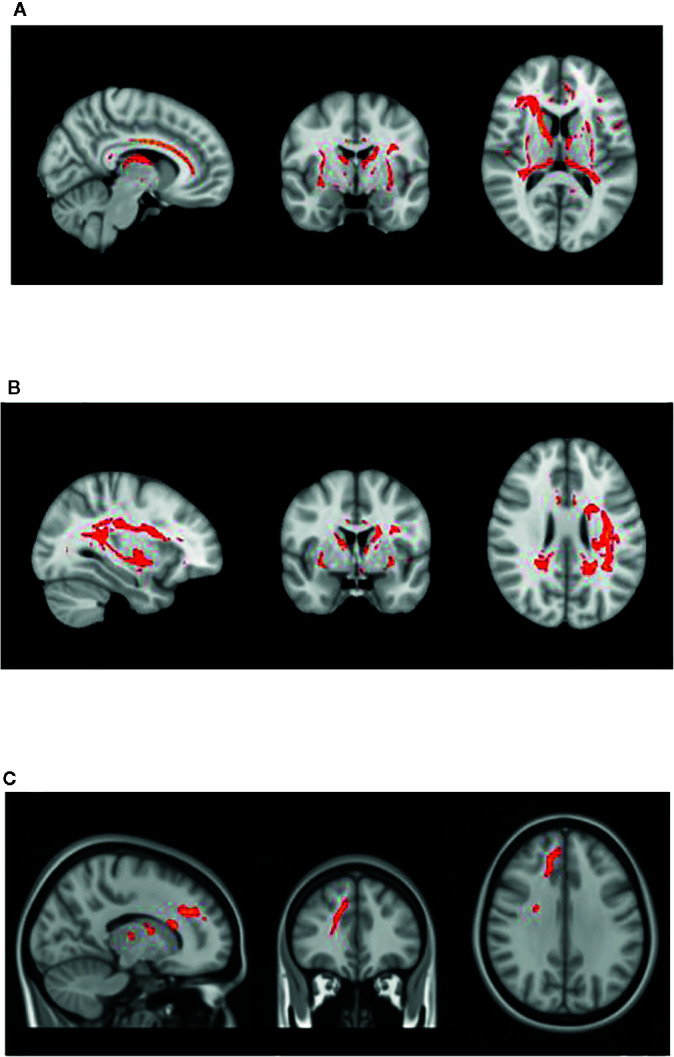
White matter tracts of interest within ‘the inhibitory control network’. **(A)** Right cingulum association fibers shown in sagittal view. Anterior corona radiata, thalamic radiations, and internal capsular projection fibers in coronal view. Inferior fronto-occipital (IFOF), IFG, and STG white matter association fibers shown in axial view. **(B)** Left Superior longitudinal (SLF) association fibers are shown in sagittal and axial views. Corona radiata and internal capsular fibers in coronal view. Cingulum tracts are seen in coronal and axial views. **(C)** Tracts connecting BA 8 and BA 6 to caudate seeds from the reaction inhibition seeds. Right SLF is shown in all orientations, while posterior corona radiata fibers are shown in coronal view, and middle frontal gyrus white matter in axial view.

**Figure 1** displays the tractography results depicting the network of tracts emanating from the seeds detailed in **Table 2**, which we have termed ‘Inhibitory Control Network’, incorporating tracts from seeds of both prospective withholding (rows A and B of **Figure 1**) and reactive inhibition phases (row C of **Figure 1**) of the f-MRI difference map, as well as the tracts between these and the needed waypoints. Images are oriented as per radiological convention (left side of the brain displayed on the right). In the right hemisphere, tracts were found between the following: inferior frontal gyrus (IFG) to superior temporal gyrus (STG), STG to posterior cingulate, posterior cingulate to IFG, posterior cingulate to the anterior cingulate cortex (ACC), IFG to caudate, and caudate to posterior cingulate. Connectivity did not occur as predicted between ACC to IFG. The association tract identified was the right inferior fronto-occipital fasciculus (IFOF) connecting IFG, STG, caudate, and posterior cingulate nodes. Although there was no direct connectivity between right IFG and ACC seeds, the IFOF connected IFG to the posterior cingulate waypoint, and the posterior cingulate connected to the right ACC *via* the cingulum. In the left hemisphere, valid tracts were found from insula to caudate, insula to inferior parietal (IFP), insula to middle temporal lobe (MTL), insula to posterior cingulate, ACC to caudate, caudate to posterior cingulate, caudate to MTL, and caudate to IFP. Connectivity was not found between insula and ACC. The majority of the association tracts in the left hemisphere belonged to left superior longitudinal fasciculus (SLF), left IFOF, and cingulum. The SLF tract connected insula, IFP, and MTL nodes, whereas the IFOF connected insula and posterior cingulate. Finally, the SLF tract was also found to connect the reactive inhibition seeds, these being, right middle frontal gyrus to caudate and right medial frontal gyrus to caudate.

**Table 2:** Seeds for tractography.

	**Coordinates (MNI)**	**Functional Network Designation**
**Prospective withholding phase:**		
**Right IFG/BA 47**	15, 28, 31	Ventral Attentional network
**Right ACC/medial prefrontal cortex/BA 32**	18, 3,45	Default Mode Network
**Right STG/BA 22**	16, 45, -19	Default Mode Network
**Left Insula/BA 13**	12, -32, -2	Ventral Attentional Network
**Left ACC/medial prefrontal cortex/BA 32**	18, 0, 46	Default Mode Network
**Left Inferior Parietal/BA 40**	7, -27, -35	Dorsal Attentional Network
**Left Middle Temporal Lobe (MTL)**	13, -42, -50	Default Mode Network
**Reactive inhibition phase:**		
**Right medial frontal gyrus/BA 8**	19, 4, 47	Default Mode Network
**Right middle frontal gyrus/BA 6**	25, 17, -13	Dorsal Attentional Network
**Waypoints:**		
**Right Caudate** **Left Caudate**	7, 11, 16 1, -3. 5	Striatum
**Right Posterior Cingulate** **Left Posterior Cingulate**	1, 5, -45 1, -15, -45	Default Mode Network

IFG, inferior frontal gyrus; ACC, Anterior Cingulate Cortex; STG, superior temporal gyrus; BA, Brodmann Area. With the exception of the two waypoints, all regions were derived directly from the f-MRI brain activation maps contrasting ADHD vs. Controls from our previous study (30), both in the prospective withholding and reactive inhibition phases of the SST. Within the waypoints, there was actual activation but no statistical differences in BOLD-Blood Oxygen Level Dependent activation between ADHD vs. Controls in any phase of the SST. For further characterization, the last column provides the identified functional network associated with the cortical seed region. This was done by importing the seeds into a functional connectivity MRI map of the human cerebral cortex, 7-network model, courtesy of Yeo et al. (39).

### White Matter Integrity Differences and Correlations With Symptom Severity and SSRT

Within the inhibitory control network, there were some white matter integrity differences (i.e. in FA) in specific areas, detailed in **Table 3**. These findings and the correlations between white matter indices (e.g. FA, RD) versus behavioral measures did not pass the multiple-comparison correction, thus represent trend differences statistically. There were no significant differences between ADHD versus controls in the secondary white integrity measures (i.e. RD, AD).

**Table 3:** Inhibitory network tracts showing fractional anisotropy changes.

Seed pair for tractography (white matter association tract)	Mean FA	Display
right IFG-STG (IFOF)	ctl= 0.39 ADHD= 0.41 P=0.09	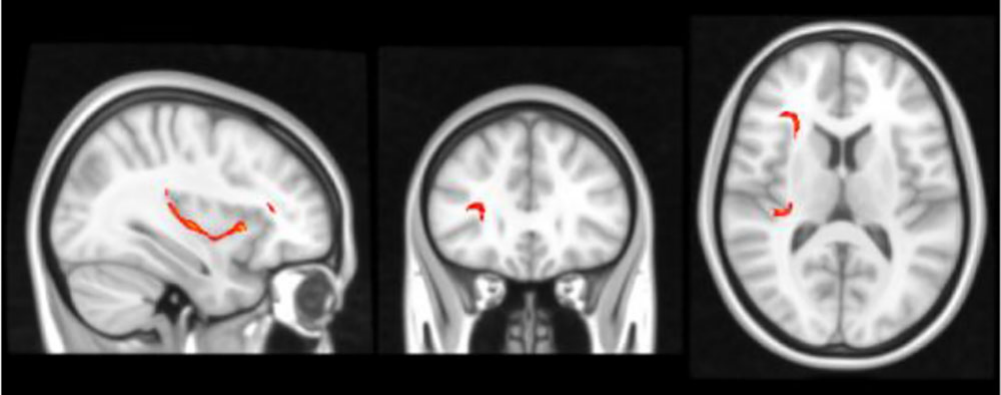
right IFG-posterior cingulate (IFOF, anterior thalamic radiations)	ctl= 0.43 ADHD=0.46 P=0.01	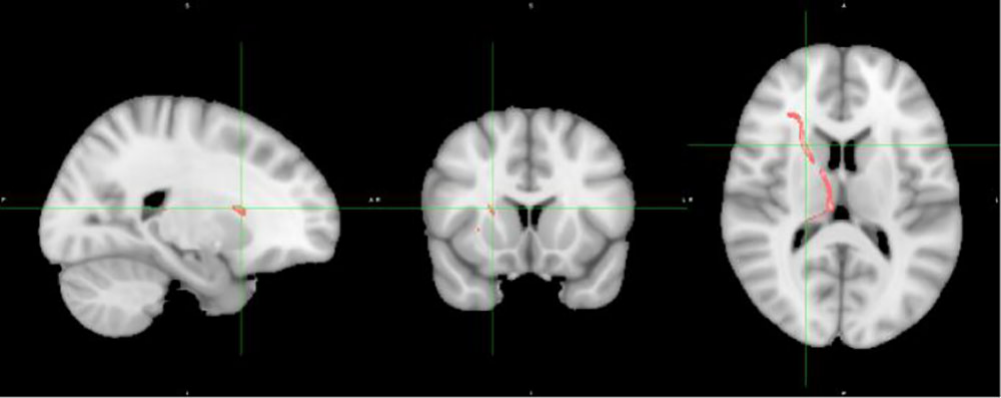
right ACC-posterior cingulate (cingulum)	ctl= 0.41 ADHD= 0.44 P=0.08	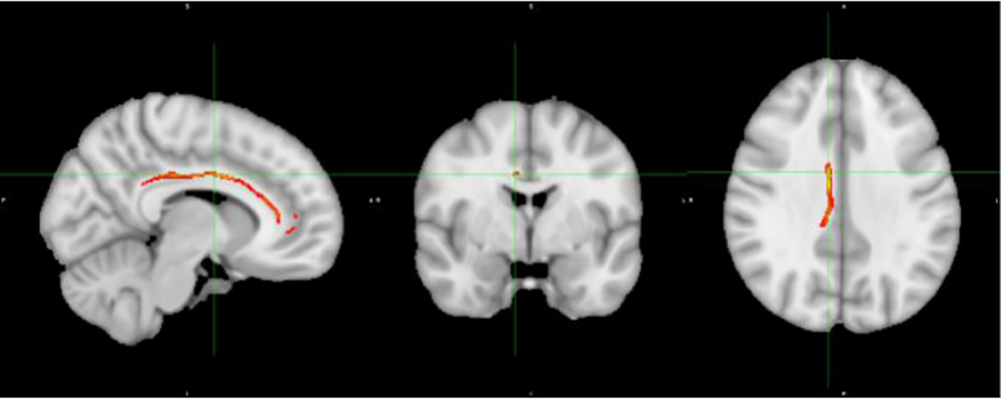
left MTL-posterior cingulate (left SLF)	ctl=0.44 ADHD= 0.47 P=0.024	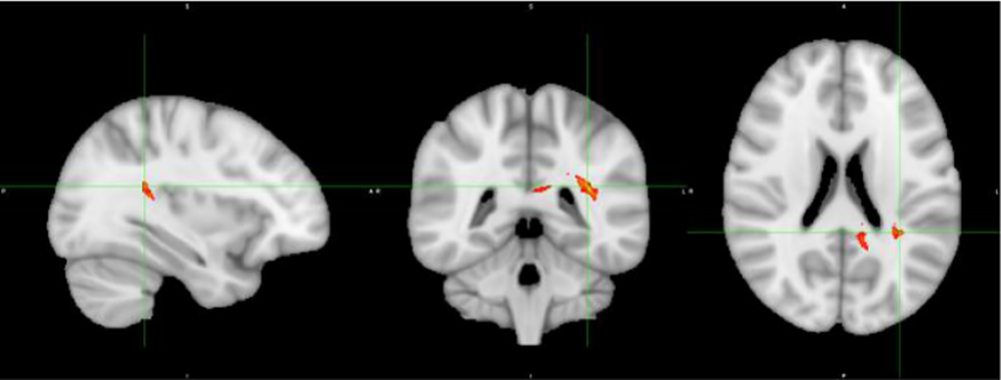

Tracts of the inhibitory network with trend differences in FA, Ctl, control.

For correlations involving inhibitory control (i.e. SSRT), we excluded the four subjects who could not withhold their ADHD medication 24 hours before study day. The mean SSRT of these four medicated children was 265 ms, which was significantly quicker (p= 0.058) compared to the rest of the group, whose mean was 408 ms ±170 ms. Notable in the medication status was that more than half of the ADHD group was not medicated for the prior six months, often due to the fact that it was their first diagnostic assessment for ADHD. We did perform t-testing to contrast if the unmedicated differed from the medicated subjects in terms of white matter indices, SWAN scores, and SSRTs, and our findings were negative. Thus, the 6-month medication status did not impact our results. Finally, given the sex differences between the groups, it was added as a co-variate in an ANOVA to assess impact and was found to not be significant.

**Figure 2:**
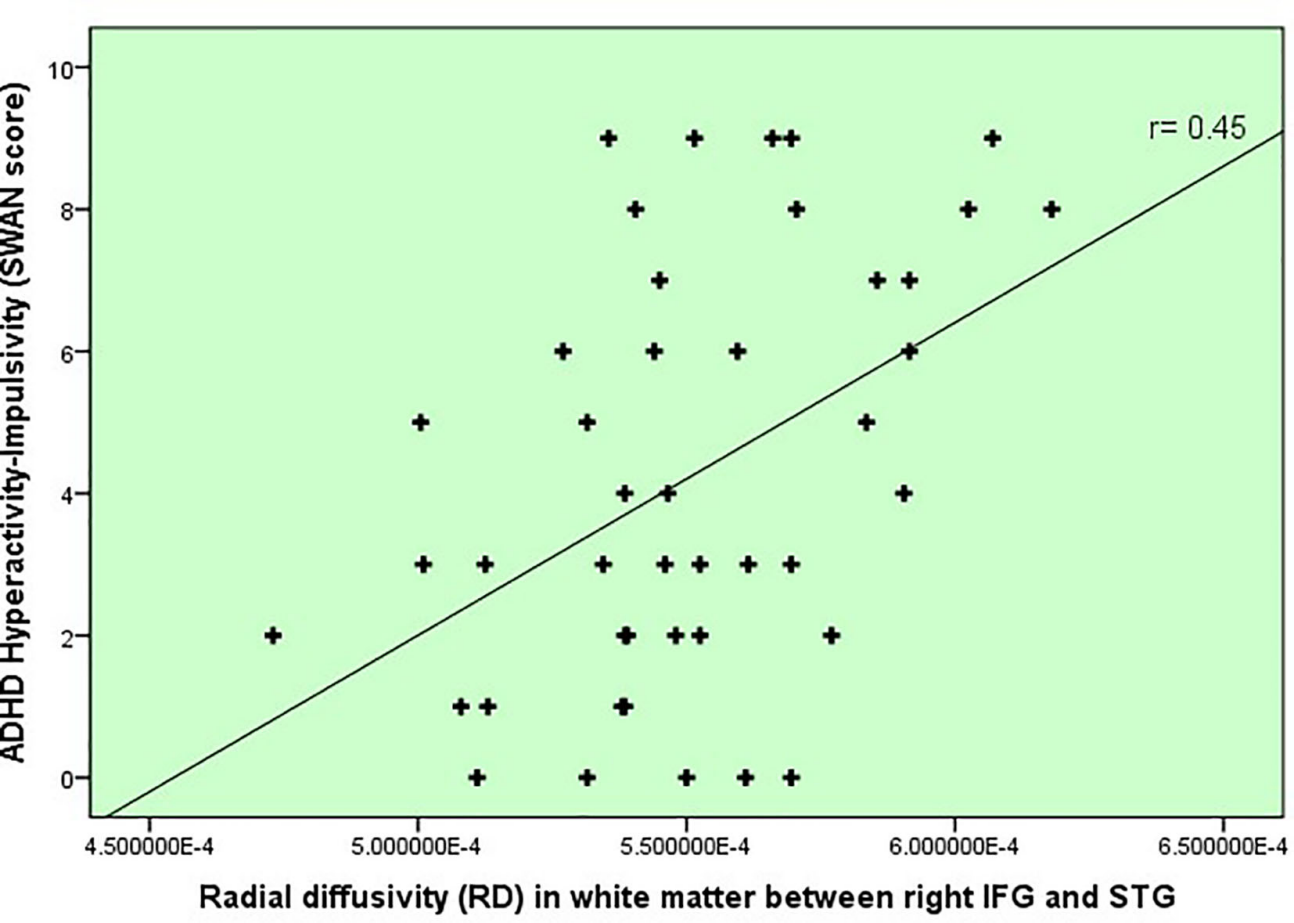
Severity of ADHD symptoms versus white matter disruption within fronto-temporal IFOF.

In terms of inhibitory control and ADHD severity, we found that poorer inhibitory control (i.e. slower SSRTs) correlated with the degree of hyperactivity-impulsivity SWAN scale score (r= .38, p=.007), rather than the inattention score (r=0.19, p=.17). This finding subsequently led us to correlate white matter changes in our **Table 3**’s tracts of interest with hyperactivity severity and found that radial diffusivity (RD) in IFOF white matter between IFG and STG correlated with hyperactivity-impulsivity scores (r = 0.45, p=0.002, uncorrected), displayed in **Figure 2**. Because age did correlate with the hyperactivity measure and thus represented a potential confounder (r= -0.38, p=.007), a linear regression was performed to test the impact of age onto the correlation between RD and hyperactivity, and showed no impact.

**Figure 3:**
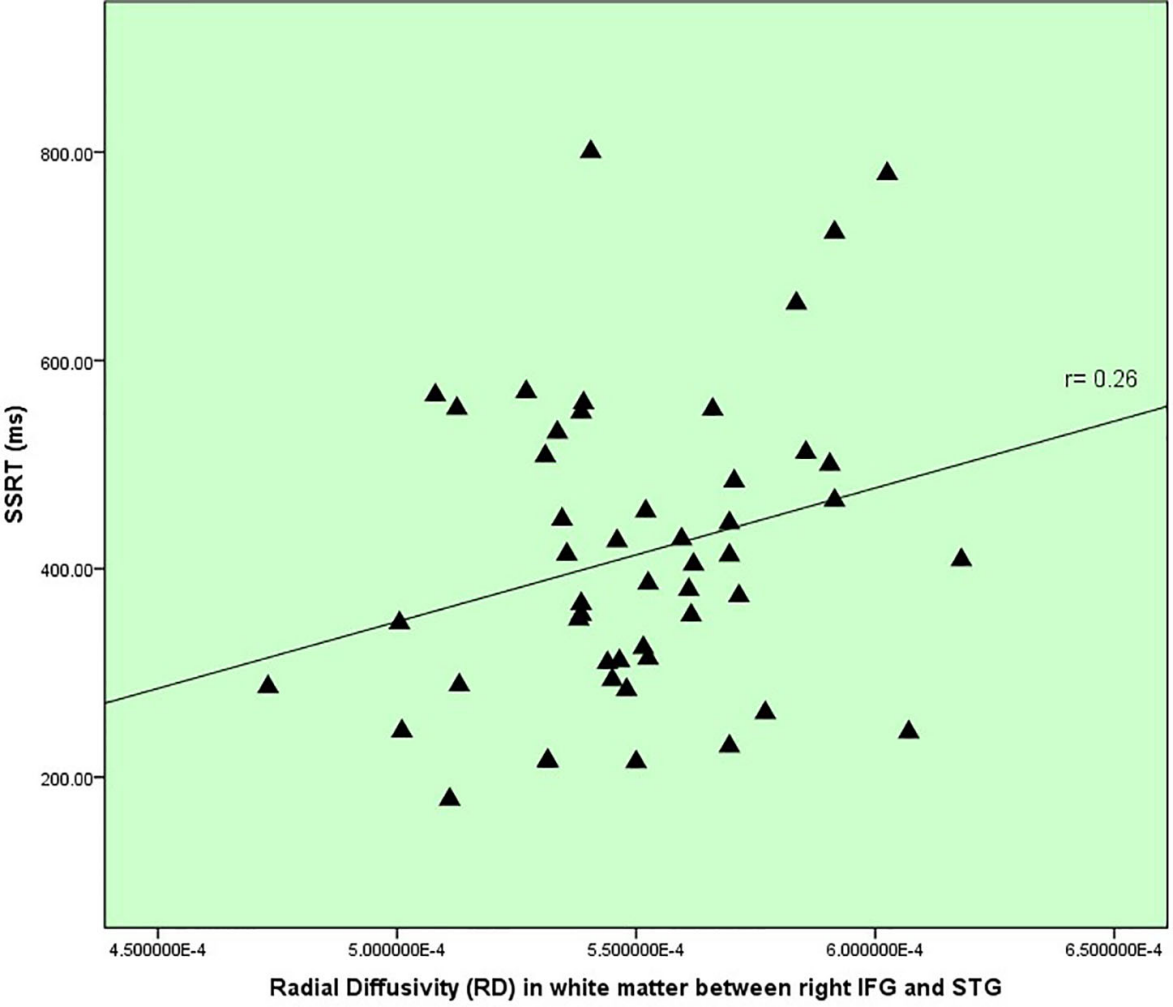
Inhibitory control versus white matter disruption in fronto-temporal IFOF.

In terms of inhibitory control and white matter changes detailed in **Table 3**, there was one correlation between RD within IFG to STG (IFOF) white matter and SSRT (r=0.26, p=.073, uncorrected), displayed in **Figure 3**, which represented a replication from the previous pilot study, discussed later. There was no correlation between age and RD within this tract.

## Discussion

In this study, we triangulated brain structural and functional changes with a neurocognitive marker of ADHD, poor inhibitory control. We isolated white matter tracts emanating specifically from seed regions imported from our previous f-MRI study, during which inhibitory control performance was tested and contrasted in ADHD youth versus healthy controls using the Stop Signal Task (30). The tracts belonged mainly to specific subsections of three association pathways: the inferior fronto-occipital fasciculus (IFOF), cingulum, and superior longitudinal fasciculus (SLF). Within our study, structural connectivity occurred between regions that are themselves implicated in very different functional networks (detailed in **Table 2**), such as the ventral attentional network (e.g. IFG, insula nodes) and default mode network-DMN (e.g. ACC, posterior cingulate, STG, and MTL nodes). Trend white matter disruptions occurred between right IFG and right STG, right IFG to right posterior cingulate, from right posterior cingulate to right ACC, and between left MTL to left posterior cingulate **(** **Table 3** **).** Interestingly, although near statistical significance, IFG to STG white matter differences between ADHD and controls replicated our previous pilot work findings (31). Also replicated from this pilot work was the association between higher radial diffusivity (RD) in this tract and degree of inhibitory control deficit (i.e. slower SSRTs). Finally, ADHD symptom severity (i.e. hyperactivity-impulsivity) also correlated with the RD of this tract.

In the case of the right IFOF tractography findings (i.e. white matter changes in IFG to STG, and IFG to posterior cingulate), one can conceptualize that it could represent a neural substrate of altered communication between ventral attentional and default mode (resting) networks, which has been proposed theoretically as a potential pathophysiological mechanism for poor inhibitory control in ADHD (30, 56). A recent study of the persistence of ADHD brain changes into adulthood reinforces this notion by finding the highest degree of aberrant inter-network connectivity occurring between DMN and ventral attentional and dorsal attentional networks (57). More specifically, in our F-MRI study, from which the tractography seeds were imported, there was decreased functional activation in IFG and increased activation in the ACC, which could have suggested a failed de-activation of the DMN (30). The current findings add to this notion by providing a brain structural substrate, that is, altered structural connectivity between the ACC and IFG through the posterior cingulate.

Our finding of IFOF involvement in inhibitory control is in line with previous studies. In healthy adults, whole-brain FA and RD correlated specifically with SSRT and not overall reaction time, and this was significant within the right IFOF and other specific regions like the right frontal gyrus (58). In our study, we also found no correlation between overall reaction time and white matter measures in this tract, as well as with any other tract within the network. In another adult ADHD study, SSRT as measured by an alternate task, the Cambridge Neuropsychological Test, correlated specifically with a white matter volume disruption within the posterior portion of the right IFOF (59), supporting the role of the IFOF in inhibitory control.

The IFOF and other white matter tracts of interest found (**Figure 1**) are also interesting from a neurodevelopmental perspective. Brain structural changes in ADHD involve broad delayed maturation of cortical thickness and of cortical surface area specifically in frontal, temporal, and parietal lobes, as well as subcortical structures (60–62). In the case of the cortex, those delays are most pronounced within anterior regions of the prefrontal cortex on the right, which would include the IFG, as well as within temporal regions including the STG (superior temporal gyrus) and MTG (middle temporal gyrus) (60). Altogether these regions are highly implicated in the current study’s inhibitory control network findings. Furthermore, a recent adult study contrasting brain connectivity in persistent versus recovered ADHD revealed increased inter-connectivity between DMN and other networks, and less intra-connectivity within the DMN itself in persistent ADHD. The DMN’s atypical connectivity occurred most prominently in left precuneus and bilateral posterior cingulate (57). The latter was used as a waypoint node in our study and revealed altered FA between right IFG to posterior cingulate, right ACC to posterior cingulate, and left MTL to posterior cingulate.

Although still debated, altered FA is an indicator of overall disruption in white matter structure, while increased RD and AD provide more direct indices of disturbed myelination (34, 63) and axonal structure (35) respectively. The FA was increased in the ADHD group for all tracts of interest (**Table 3**), without alteration in other indices including RD and AD, which suggests potentially undisturbed myelination, but persistence or inappropriate connectivity between these structures instead. It is plausible that given that ADHD cortical maturation is delayed, that white matter development delays follow the same trend. The coherence between cortical and white matter maturational mechanisms has only very recently been studied in typically developing youth and show utmost complexity. Coherence between cortical thinning and increased FA occurred only in the frontal lobe, and regionally at different rates (64). The importance of the developmental time window, specific brain region, and directionality of white matter indices was demonstrated in a reading abilities study, where reading performance increased with FA in early years within dorsal tracts, but after 10 years of age, this correlation ended and shifted to a correlation of diminished reading performance with FA increases within the right ventral tracts including IFOF (65). Past DTI studies of ADHD show that the directionality of white matter integrity indices (e.g. both increases and decreases in FA) vary significantly between studies, even within similar tracts of interest, and it also includes findings of no differences in FA in major association tracts (9, 66, 67). A recent study divided combined-type ADHD from inattentive-type ADHD before comparing to controls, and found increases in FA in specific pathways for the subtypes, including higher FA in cingulum bundle for the combined-typed ADHD group (68). In our study, the vast majority of subjects were combined-type and also showed increased FA in this tract.

Another interesting finding in this study was the lack of differences in white matter indices within the fronto-striatal seeds of the prospective withholding and reactive inhibition phases. This system has been a focus of past brain function studies of SST performance in neurotypical adults as well as in ADHD pathophysiology (4, 69). However, our findings suggest that it may not be related directly to the inhibitory control deficit in ADHD, given the lack of correlation between SSRT and white matter integrity, and the previously documented lack of differences between ADHD and control groups in brain activity during SST performance within these fronto-striatal regions (30). Interestingly, in Chiang’s et al. DSI study, wherein fronto-striatal white matter in ADHD youth and controls was reconstructed and correlated with output measures of an attentional task, they found an overall stronger association between this system’s integrity and performance in controls rather than the ADHD group (70).

In terms of study limitations, a broader control sample, with more equivalent sex ratios, and with SST performance data, could help further elucidate the relationship between SSRT and white matter indices as well as the white matter contrasts, which remained trend differences due to the multiple-comparison effect. It should be noted that although not directly compared to a control sample, the extent of the inhibitory control deficit as measured by the SSRT within our ADHD group was pronounced and comparable to a past large community sample study correlating SSRT with ADHD symptoms (29). Another limitation of our study lies in the spatial resolution of the DTI protocol. DTI technology is exponentially improving, and only very recently it has been possible to capture not only large association tracts but intra-lobar pathways using spherical de-convolution diffusion tractography (38), requiring however extended time in the scanner.

In conclusion, the results of this experiment point to specific white matter tracts implicated in inhibitory control processing in ADHD children. These results may help direct us closer to a potential biomarker incorporating brain function, structure together with an important neurocognitive deficit. There is growing evidence that white matter changes can represent endophenotypic markers in psychiatric disorders. For example, white matter volumetric differences within the posterior portion of the right IFOF and grey matter within the right IFG have been found in adult ADHD cases and their first degree relatives, with a correlation between SSRT and degree of white matter disruption (59). Future avenues of study could include exploring the state of the inhibitory control network in other psychiatric conditions where poor inhibitory control is found such as in autism. Also, implementing pharmacologically informed seeds into a tractography study (e.g. using nodes where psychostimulants alter brain activation in ADHD patients), or using seeds informed by other cognitive deficits like altered reward processing (71), temporal processing (72), and working memory deficits (73), which together would enhance our understanding of the brain mechanisms of ADHD and drive future therapeutic targets.

## Data Availability Statement

The datasets generated for this study are available on request to the corresponding author.

## Ethics Statement

The studies involving human participants were reviewed and approved by Research Ethics Board of Hospital for Sick Children, University of Toronto. Written informed consent to participate in this study was provided by the participants’ legal guardian/next of kin.

## Author Contributions

LT: Lead of the project, including methodology, hypotheses, analyses, writing of manuscript. CH: Statistical and data analytics, including data pre-processing, tractography, statistical review, manuscript review/critique. SA: Diffusion Tensor Imaging expertise and oversight of current data set, overview of tractography, teaching of first author, manuscript review/critique. MB: Contributed the f-MRI data expertise, and provided f-MRI dataset used in initial pilot project, collaborator in current project, manuscript review/critique. DM: Diffusion Tensor Imaging teaching and guidance, senior scientist that reviewed the DTI aspect, manuscript review. EA: Lead in data collection for POND consortium, senior scientist, manuscript review/critique. JL: Expertise in Diffusion Tensor Imaging processing, tractography, senior scientist, manuscript review/critique. RS: Expertise in the application of Stop Signal Task, response inhibition and ADHD, data collection and review, senior scientist, manuscript review and critique. All authors contributed to the article and approved the submitted version.

## Funding

This research was funded by the Canadian Institute of Health Research (CIHR), MOP 82796 and the Ontario Brain Institute (OBI).

## Conflict of Interest

The authors declare that the research was conducted in the absence of any commercial or financial relationships that could be construed as a potential conflict of interest.
